# The Ubiquitin Ligase RNF34 Participates in the Peripheral Quality Control of CFTR (RNF34 Role in CFTR PeriQC)

**DOI:** 10.3389/fmolb.2022.840649

**Published:** 2022-03-09

**Authors:** Shogo Taniguchi, Yukiko Ito, Hibiki Kiritani, Asuka Maruo, Ryohei Sakai, Yuji Ono, Ryosuke Fukuda, Tsukasa Okiyoneda

**Affiliations:** Department of Biomedical Sciences, School of Biological and Environmental Sciences, Kwansei Gakuin University, Sanda, Japan

**Keywords:** cystic fibrosis, CFTR, ubiquitin ligase, peripheral quality control, RFFL, RNF34, Trikafta

## Abstract

The peripheral protein quality control (periQC) system eliminates the conformationally defective cystic fibrosis transmembrane conductance regulator (CFTR), including ∆F508-CFTR, from the plasma membrane (PM) and limits the efficacy of pharmacological therapy for cystic fibrosis (CF). The ubiquitin (Ub) ligase RFFL is responsible for the chaperone-independent ubiquitination and lysosomal degradation of CFTR in the periQC. Here, we report that the Ub ligase RNF34 participates in the CFTR periQC in parallel to RFFL. An *in vitro* study reveals that RNF34 directly recognizes the CFTR NBD1 and selectively promotes the ubiquitination of unfolded proteins. RNF34 was localized in the cytoplasm and endosomes, where RFFL was equally colocalized. RNF34 ablation increased the PM density as well as the mature form of ∆F508-CFTR rescued at low temperatures. RFFL ablation, with the exception of RNF34 ablation, increased the functional PM expression of ∆F508-CFTR upon a triple combination of CFTR modulators (Trikafta) treatment by inhibiting the K63-linked polyubiquitination. Interestingly, simultaneous ablation of RNF34 and RFFL dramatically increased the functional PM ∆F508-CFTR by inhibiting the ubiquitination in the post-Golgi compartments. The CFTR-NLuc assay demonstrates that simultaneous ablation of RNF34 and RFFL dramatically inhibits the degradation of mature ∆F508-CFTR after Trikafta treatment. Therefore, these results suggest that RNF34 plays a crucial role in the CFTR periQC, especially when there is insufficient RFFL. We propose that simultaneous inhibition of RFFL and RNF34 may improve the efficacy of CFTR modulators.

## Introduction

The cystic fibrosis transmembrane conductance regulator (CFTR) is a cAMP-regulated chloride channel expressed on the plasma membrane (PM) of epithelial cells (Riordan, 2008), where its mutation gives rise to cystic fibrosis (CF), a recessive genetic disorder (Guggino, 1999). The most common ∆F508 mutation causes the misfolding in the CFTR protein which is then eliminated by a cellular protein quality control (QC) system. Most of the ∆F508-CFTR is ubiquitinated at the ER by multiple ubiquitin ligases, including CHIP (STUB1) (Meacham et al., 2001), RNF5 (Younger et al., 2006), and RNF185 (El Khouri et al., 2013). This results in ER-associated degradation (ERAD), limiting the PM expression of the partially functional channel. ∆F508-CFTR is capable of reaching the PM at either low temperatures (26°C–30°C) or in the presence of CFTR correctors, which has been used as a component of an approved drug for CF. However, ∆F508-CFTR is still eliminated from the PM by the peripheral protein QC (periQC) mechanism that limits the efficacy of CF pharmacological therapy (Okiyoneda et al., 2011). periQC facilitates the CFTR ubiquitination and lysosomal degradation of conformationally defective CFTR. Ubiquitin ligases CHIP and RFFL have been reported responsible for the CFTR ubiquitination in the periQC mechanism (Okiyoneda et al., 2010). CHIP is a chaperone-associated ubiquitin (Ub) ligase and interacts with immature and mature ∆F508-CFTR at the ER and post-Golgi compartments, respectively, through Hsc70/Hsp70 and Hsp90 complexes (Younger et al., 2004; Okiyoneda et al., 2010). In contrast, RFFL, an FYVE-like domain-containing Ub ligase, directly interacts with mature ∆F508-CFTR in the post-Golgi compartments, including endosomes and PM through its disordered regions located in the N-terminal region (Okiyoneda et al., 2018). Both CHIP and RFFL selectively bind and facilitate ubiquitination of the conformationally defective CFTR, resulting in rapid endocytosis and lysosomal degradation. With the exception of CHIP and RFFL, Ub ligases responsible for the periQC ultimately remain unknown, whereas various E3 ligases have been reported to determine the ERQC of CFTR.

RNF34 (CARP1) is a caspase 8/10-associated Ub ligase with high homology to RFFL (McDonald and El-Deiry, 2004; Tibbetts et al., 2004). Both RNF34 and RFFL are highly expressed in cancer cells and have a common function in the degradation of p53 (Sasaki et al., 2004; Yang et al., 2007). Moreover, RNF34 and RFFL have a shared function in targeting RIP1 and regulating TNF-induced NF-κB activation (Liao et al., 2008). BioID analysis also suggests the proximal localization of RNF34 with RFFL (Sakai et al., 2019). Thus, it is hypothesized that RNF34 may be involved in the periQC of CFTR in addition to RFFL.

Here, we report that RNF34 functions for the periQC of CFTR. This is shown by RNF34 selectively interacting with unfolded proteins and directly facilitating their ubiquitination. Like RFFL, RNF34 is localized in endosomal compartments. Discussed in further detail, RNF34 ablation minimally affected the ∆F508-CFTR upon Trikafta treatment. However, simultaneous ablation of RNF34 and RFFL dramatically increased the functional expression of ∆F508-CFTR after Trikafta treatment by inhibiting the K63-linked polyubiquitination and degradation in the post-Golgi compartments. Thus, RNF34 determines the CFTR periQC, and simultaneous inhibition of RFFL and RNF34 may further improve the efficacy of CFTR modulators.

## Materials and methods

The Materials and Methods are provided in the Supplementary material for this paper.

## Results

### RNF34 directly ubiquitinates the unfolded proteins

**FIGURE 1:**
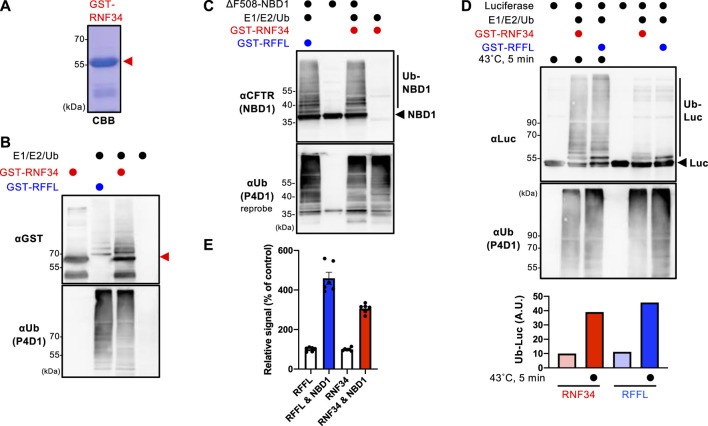
RNF34 directly ubiquitinates the unfolded proteins *in vitro*. **(A)** Purified GST-RNF34 (arrowhead) was confirmed by SDS-PAGE and visualized with Coomassie brilliant blue (CBB) staining. **(B)** Auto-ubiquitination of GST-RNF34 or GST-RFFL in the presence of E1, E2, and Ub was analyzed by Western blotting. The arrowhead shows the unmodified GST-RNF34. **(C)** *In vitro* ubiquitination of unfolded ΔF508-NBD1 by GST-RNF34 or GST-RFFL. **(D)** *In vitro* ubiquitination of Luciferase (Luc) thermally unfolded (43°C, 5 min) by GST-RNF34 or GST-RFFL. Ub-Luc level was quantitated by densitometry. **(E)** AlphaLISA detected direct interaction between GST-RFFL or GST-RNF34 and His-sumo-ΔF508-NBD1. Data represent means ± SE (n = 6).

The GST-RNF34 protein was purified from *E. coli* to characterize the function (Figure 1A). *In vitro* ubiquitination assays confirmed that GST-RNF34 is active as it induced auto-ubiquitination in the presence of E1 and E2 enzymes (Figure 1B). Like RFFL (Okiyoneda et al., 2018), GST-RNF34 ubiquitinated the ΔF508-NBD1 (Figure 1C) and luciferase (Luc) *in vitro* (Figure 1D). Moreover, thermal unfolding (43°C, 5 min) augmented the RNF34-mediated Luc ubiquitination (Figure 1D), indicating that RNF34 selectively facilitates the ubiquitination of conformationally defective proteins. The direct binding of GST-RNF34 to His-sumo-ΔF508-NBD1 was evaluated by AlphaLISA. In addition to GST-RFFL, GST-RNF34 is directly bound to ΔF508-NBD1 (Figure 1E). These results demonstrate that RNF34 directly interacts with the unfolded proteins and selectively facilitates ubiquitination.

### RNF34 Ablation Increased the PM Levels of Low-Temperature Rescued ∆F508-CFTR

**FIGURE 2:**
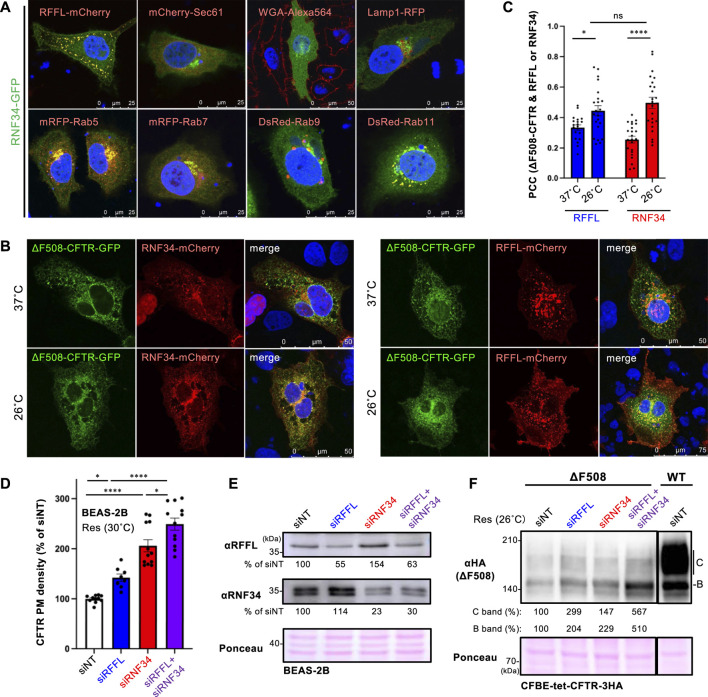
RNF34 locates in endosomes with RFFL and limits the PM levels of low-temperature rescued ∆F508-CFTR. **(A)** Cellular localization of RNF34-GFP in HeLa-∆F508 CFTR-3HA cells was analyzed with either the co-transfected organelle markers indicated or a WGA-Alexa Fluor 564 staining. The nucleus was stained with DAPI. **(B,C)** Colocalization of RNF34-mCherry or RFFL-mCherry with ∆F508-CFTR-GFP in COS7 cells were measured by determining the Pearson’s correlation coefficient (PCC, *n* = 20–23 cells). Statistical significance was assessed by two-tailed paired Student’s t-test. Cells were incubated at either 37°C or 26°C for 2 days, followed by a 1-h incubation at 37°C before fixation. **(D,E)** PM levels of low-temperature rescued ∆F508-CFTR-HiBiT in BEAS-2B cells transfected with siRNA are (50 nM each) indicated (*n* = 8–12). Statistical significance was assessed by one-way ANOVA. Western blotting confirmed the RNF34, and RFFL KD and the KD efficiency were quantitated by densitometry **(E)**. Ponceau-S staining was used as the loading control. **(F)** Expression of low-temperature rescued ∆F508-CFTR-3HA in CFBE cells transfected with siRNA are indicated. Ponceau-S staining was used as the loading control. Data represent means ± SE. ns, not significant, *p < .05, ****p < .001.

To examine the cellular localization of RNF34, RNF34-GFP was co-expressed with organelle markers. Confocal microscopy analysis revealed that RNF34-GFP was localized in the cytoplasm and punctate structures where RFFL-mCherry was colocalized (Figure 2A, Supplementary Figure S1A). Similar to the RFFL, RNF34-GFP was colocalized with Rab5 (early endosome), Rab7 (late endosome), and Rab11 (recycling endosome), indicating the endosome localization as shown before (Jin et al., 2014) (Figure 2A, Supplementary Figure S1A). As expected, RNF34-mCherry and RFFL-mCherry were partially colocalized with ∆F508-CFTR-GFP, and low-temperature incubation increased the colocalization measured by the Pearson’s correlation coefficient (PCC) (Figures 2B,C). These results suggest that RNF34 may participate in the CFTR periQC in addition to RFFL.

Next, we examined the effect of RNF34 knockdown (KD) on the ∆F508-CFTR rescued by low-temperature incubation. Similar to RFFL, RNF34 KD significantly increased the PM levels of low-temperature rescued ∆F508-CFTR-HiBiT in BEAS2-B cells (Figures 2D,E). In support of this finding, a Western blot analysis showed that KD of RNF34 or RFFL increased the immature and mature form of ∆F508-CFTR-3HA in CFBE cells (Figure 2F). Interestingly, the RFFL/RNF34 double KD further increased both immature and mature forms and PM levels of ∆F508-CFTR compared to every single KD (Figures 2D,F). These results suggest that RNF34 participates in the periQC in parallel to RFFL, limiting the PM levels of low-temperature rescued ∆F508-CFTR.

### Simultaneous Inhibition of RNF34 and RFFL Dramatically Enhances the Efficacy of CFTR Modulators

**FIGURE 3:**
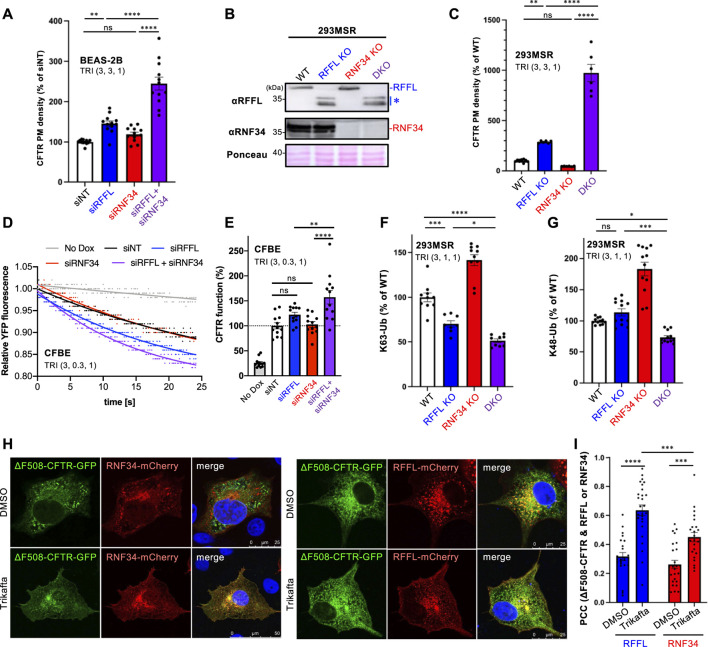
Simultaneous ablation of RNF34 and RFFL synergistically enhances the Trikafta effect on the PM ∆F508-CFTR expression and function. **(A)** PM density of ∆F508-CFTR-HiBiT stably expressed in BEAS-2B cells transfected with siRNA are indicated (50 nM each). Cells were treated with Trikafta (3 µM VX-661, 3 µM VX-445, 1 µM VX-770) at 37°C for 48 h (*n* = 12). **(B)** Western blotting confirms the RNF34 and RFFL KO in 293MSR cells. Asterisk shows N-terminus truncated non-functional RFFL. Ponceau-S staining was used as the loading control. **(C)** PM density of ∆F508-CFTR-HRP transiently expressed in RNF34 and RFFL KO cells treated with Trikafta (3 µM VX-661, 3 µM VX-445, 1 µM VX-770) at 37°C for 24 h (*n* = 6). **(D,E)** Representative traces **(D)** and quantification of the initial YFP quenching rate **(E)** of ∆F508-CFTR-3HA function in CFBE siRNA transfected and pretreated with Trikafta (3 µM VX-661, 0.3 µM VX-445, 1 µM VX-770) at 37°C for 48 h (*n* = 12). **(F,G)** K63- **(F)** and K48-linked poly-ubiquitination **(G)** of the mature HBH-∆F508-CFTR in the KO cells were quantified by Ub ELISA using a Ub linkage-specific antibody. Trikafta (3 µM VX-661, 1 µM VX-445, 1 µM VX-770) was treated at 37°C for 48 h (*n* = 6). **(H,I)** Colocalization of RNF34-mCherry or RFFL-mCherry with ∆F508-CFTR-GFP in COS7 cells treated with Trikafta (3 µM VX-661, 3 µM VX-445, 1 µM VX-770) for 48 h is analyzed as shown in Figures 2B,C (*n* = 23–27 cells). Statistical significance was assessed by either one-way ANOVA **(A,C,E,F,G)** or two-tailed paired Student’s *t*-test **(I)**. Data represent mean ± SE. *p < .05, **p < .01, ***p < .005, ****p < .001, ns, not significant.

Understanding that the periQC system limits the efficacy of CFTR modulators (Okiyoneda et al., 2018); next, we evaluated the KD of RFFL and RNF34 on PM levels of ∆F508-CFTR upon treatment with the triple combination of CFTR modulators named Trikafta (VX-661/VX-445/VX-770). Trikafta is the newest FDA-approved drug that robustly corrects the misfolding and functional PM expression of ∆F508-CFTR (Keating et al., 2018; Veit et al., 2020; Baatallah et al., 2021). As expected, the RFFL KD significantly increased the ∆F508-CFTR PM levels upon treatment with Trikafta in BEAS-2B (Figure 3A) and CFBE cells (Supplementary Figure S2A). Although the RNF34 KD had minimal effect, the RFFL/RNF34 double KD significantly increased the ∆F508-CFTR PM levels more than the RFFL KD (Figure 3A, Supplementary Figure S2A) alone. To clarify this KD effect, RNF34 knockout (KO) and RFFL/RNF34 double KO (DKO) 293MSR cells were generated by the CRISPR-Cas9 system such that RFFL KO cells were established previously (Sakai et al., 2019). Western blotting confirmed the abolished endogenous expression of RNF34 and full-length RFFL as previously reported (Sakai et al., 2019) (Figure 3B). In the RFFL KO cells where the exon two containing the start codon was deleted, the N-terminal truncated RFFL (presumably deleting 1–71 aa) was expressed using a downstream cryptic start codon (Sakai et al., 2019) (Figure 3B). As expected, the RFFL KO, in contrast to the RNF34 KO, increased the PM levels of ∆F508-CFTR-HRP transiently expressed in 293MSR cells after the Trikafta treatment (Figure 3C). Moreover, the RFFL/RNF34 DKO dramatically increased the PM levels (Figure 3C). The halide-sensitive YFP quenching assay revealed that the RFFL/RNF34 double KD significantly increased the functional ∆F508-CFTR channel induced by Trikafta (Figures 3D,E). Additionally, Ub ELISA showed that the RFFL KO significantly reduced the K63-linked polyubiquitination of mature ∆F508-CFTR, but not RNF34 KO upon Trikafta treatment (Figure 3F). The RNF34/RFFL DKO reduced the K63-linked polyubiquitination more than the RFFL KO alone (Figure 3F). The K48-linked polyubiquitination was decreased significantly by the RFFL/RNF34 DKO, but KOs of neither RFFL nor RNF34 (Figure 3G) individually. These results suggest that RFFL primarily facilitates the K63-linked poly-ubiquitination of mature ∆F508-CFTRs in the post-Golgi compartments upon Trikafta treatment while RNF34 functions only when RFFL function is insufficient. Unexpectedly, the RNF34 KO increased the CFTR K63- and K48-linked polyubiquitination (Figures 3F,G). We found the increased RFFL mRNA and protein in RNF34 KO cells, presumably leading to the increased CFTR ubiquitination (Supplementary Figures S2B,C). Increased RNF34 mRNA and protein levels after RFFL ablation were also observed (Supplementary Figures S2D,E). Similar to the low-temperature rescue, the Trikafta treatment facilitated the colocalization of ∆F508-CFTR with RFFL and RNF34 (Figures 3H,I). Interestingly, RFFL was significantly co-localized with ∆F508-CFTR than with RNF34 after Trikafta treatment (Figures 3H,I). This result also supports RFFL primary functions in the CFTR periQC upon Trikafta treatment.

### A CFTR-NLuc Degradation Assay Clarified the Effect of RNF34 and RFFL on the CFTR periQC

**FIGURE 4:**
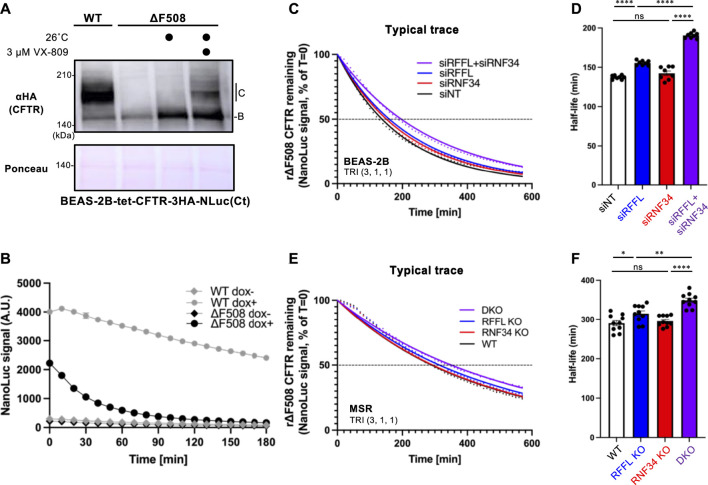
Simultaneous ablation of RNF34 and RFFL synergistically stabilizes the mature ∆F508-CFTR induced by Trikafta treatment. **(A)** Expression of WT- and ∆F508-CFTR-3HA-NLuc in BEAS-2B cells. ∆F508-CFTR was rescued by 26°C incubation for 2 days with or without 3 µM VX-809. Ponceau-S staining was used as the loading control. **(B)** The WT- or ∆F508-CFTR-NLuc level in BEAS-2B cells was monitored in real-time using Endurazine substrates following an 100 μg/ml CHX treatment at 37°C. The CFTR expression was induced by a 1-μg/ml Dox treatment for 2 days (*n* = 6). **(C,D)** Representative traces of mature ∆F508-CFTR-NLuc elimination in BEAS-2B cells transfected with siRNA are indicated **(C)**. Cells were pre-treated with Trikafta (3 µM VX-661, 1 µM VX-445, 1 µM VX-770) at 37°C for 24 h. The half-life of mature ∆F508-CFTR-NLuc was quantified [**(D)**, *n* = 8]. **(E,F)** The mature ∆F508-CFTR-NLuc elimination **(E)** and half-life **(F)** were measured in the RNF34 and RFFL KO cells as **(C,D)** (*n* = 10). Statistical significance was assessed by one-way ANOVA. Data represent mean ± SE. *p < .05, **p < .01, ****p < .001, ns, not significant.

To quantitatively measure the effect of RNF34 and RFFL on CFTR degradation, we have developed an assay which accurately quantifies the kinetic CFTR degradation in live cells. NanoLuc (NLuc) was fused with CFTR in the C-terminus (CFTR-NLuc) and stably expressed in BEAS-2B tet-on cells. As expected, WT CFTR-NLuc was expressed in mature and immature forms while ∆F508-CFTR-NLuc was only expressed in its immature form (Figure 4A). The NLuc fusion might slightly destabilize the ∆F508-CFTR as the low temperature incubation may not have been sufficient to induce the maturation (Figure 4A). Corrector VX-809 treatment at low temperature induced the maturation of ∆F508-CFTR-NLuc (Figure 4A). The continuous luminescent measurement in the live cells showed that ∆F508-CFTR-NLuc was largely eliminated in 90 min by ERAD (Figure 4B). In contrast, WT CFTR-NLuc was completely stable as expected (Figure 4B). Next, we measured the degradation of mature ∆F508-CFTR-NLuc induced by Trikafta treatment. The immature form was minimized by cycloheximide (CHX) treatment for 3 h during the NLuc substrate loading. The luminescent measurement revealed that the mature ∆F508-CFTR-NLuc induced by Trikafta treatment continuously decreased, and its half-life was ∼140 min in the siNT-transfected cells (Figures 4C,D). The RFFL KD, in contrast to the RNF34 KD, significantly increased the half-life of mature ∆F508-CFTR-NLuc (Figures 4C,D). As expected, the RFFL/RNF34 double KD further extended the half-life compared to the RFFL KD (Figures 4C,D). Similar phenotypes were observed in the KO cells (Figures 4E,F). These results strongly suggest that RNF34 functions in the periQC to facilitate the ∆F508-CFTR degradation in the post-Golgi compartments when the RFFL function is insufficient.

## Discussion

This study demonstrates that RNF34 is involved in the CFTR periQC. *In vitro* experiments revealed that, like RFFL, RNF34 directly interacts with unfolded NBD1 and selectively ubiquitinates the unfolded NBD1 and Luc. Cellular localization analysis demonstrated that RNF34 was colocalized with RFFL in the endosomal compartments consistent with their proximal localization (Sakai et al., 2019). Moreover, RNF34 was more colocalized with ∆F508-CFTR after low-temperature incubations or Trikafta treatments. These results suggest that RNF34, in addition to RFFL, facilitates the ubiquitination of CFTR in the endosomal compartments. Although its precise mechanism remains unclear, RNF34 could be capable of recognizing the unfolded proteins through its N-terminal region, including the disordered region and FYVE-like domain with high homology to RFFL (Tibbetts et al., 2004). Given that RNF34 ubiquitinated the unfolded Luc *in vitro*, RNF34 may ubiquitinate not only the unfolded CFTR but also other unfolded proteins located in the endosomal compartments. Although the involvement of the periQC mechanism remains unclear, it has been reported that endosomal RNF34 facilitates the GABA_A_ receptor degradation by ubiquitinating the *γ*2 subunit (Jin et al., 2014).

In this study, we found that the RNF34 KD increased the PM expression and mature form of low-temperature rescued ∆F508-CFTRs, a similar phenotype observed in the RFFL KD. In comparison, unlike RFFL, RNF34 ablation failed to increase the functional PM expression of ∆F508-CFTR upon Trikafta treatment. The Trikafta treatment seems to completely correct the ∆F508-CFTR misfolding, including NBD1 and the NBD1-MSD interface (Veit et al., 2020). Low-temperature incubation partially restores the CFTR misfolding, most likely only in the NBD1 instability (Lukacs and Verkman, 2012). Thus, RNF34 may selectively recognize the more severely unfolded CFTR, such as the low-temperature rescued ∆F508-CFTR in the endosomal compartments. On the other hand, RFFL ablation led to increased PM expression of ∆F508-CFTR rescued by low-temperature incubation or Trikafta treatment. Thus, RFFL may recognize the conformationally defective CFTR more sensitively than RNF34. Interestingly, we found increased RFFL expression in RNF34 KO cells. RFFL upregulation may minimize the impact of the RNF34 KO on the ∆F508-CFTR degradation as RFFL appears to have an at least partially overlapped function in the CFTR periQC. Increased RFFL expression could result in unexpectedly increased K48- and K63-linked polyubiquitination of mature ∆F508-CFTR in RNF34 KO cells. On another note, the RNF34 mRNA and protein levels also increased by the RFFL ablation. These results suggest that the expression of RFFL and RNF34 could be reciprocally regulated to maintain the periQC system.

We found that the mature ∆F508-CFTR induced by Trikafta treatment was still ubiquitinated and less stable than the WT CFTR. These results are consistent with a recent study that reported the CFTR phenotypes upon treatment of VX-661/VX-445 (Capurro et al., 2021). Additionally, RFFL ablation significantly stabilized the mature ∆F508-CFTR by reducing the K63-linked polyubiquitination and increased the functional PM expression, even after the Trikafta treatment. These results suggest that despite Trikafta robustly correcting the folding defects of ∆F508-CFTR, the periQC machinery, including RFFL, is still capable of recognizing the cell surface ∆F508-CFTR for elimination. Therefore, it can be noted that RFFL inhibition may maximize the efficacy of efficient correctors available.

RNF34 ablation failed but synergistically increased the PM levels of ∆F508-CFTR rescued by Trikafta under the RFFL KO. This result suggests that RNF34 and RFFL act through different mechanisms. Consistent with these results, the mature ∆F508-CFTR poly-ubiquitination was equally synergistically inhibited by the RFFL/RNF34 DKO. The CFTR-NLuc assay, which quantifies the kinetic CFTR degradation in live cells, clearly demonstrated that RNF34 was not essential for the unfolded CFTR degradation in the post-Golgi compartments when RFFL is active. However, when RFFL is insufficient, RNF34 plays a significant role in the periQC machinery that is crucial for CFTR degradation. We cannot exclude the possibility that RNF34 participates in the ERQC of CFTR as RNF34 ablation increases the immature ∆F508-CFTR. Future studies will be required to clarify this possibility.

In summary, we identify RNF34 as a new component of the CFTR periQC machinery. RNF34 determines the ubiquitination of unfolded CFTR in the endosomal compartments in parallel to RFFL. We propose that simultaneous inhibition of RFFL and RNF34 may facilitate the functional PM expression of ∆F508-CFTR even upon Trikafta treatment. However, future studies will be required to validate the impact on ∆F508-CFTR in CF-HBE cells where both RFFL (Okiyoneda et al., 2018) and RNF34 were expressed (Supplementary Figure S3).

## Data Availability

The raw data supporting the conclusion of this article will be made available by the authors, without undue reservation.
