# Supplementary material for: The Ubiquitin Ligase RNF34 Participates in the Peripheral Quality Control of CFTR (RNF34 Role in CFTR PeriQC)
Source: Front Mol Biosci. 2022 Mar 9;9:840649. doi: 10.3389/fmolb.2022.840649 (PMC8959631; doi:10.3389/fmolb.2022.840649)
Supplement: Supplementary file 1 [file Presentation1.pdf]

The Supplementary material

Materials and Methods

Protein purification

GST-RFFL, GST-RNF34, His_6-UBE1 (addgene #34965), His_6-sumo-UbcH5c and His_6-sumo-NBD1-ΔF1S were expressed in a BL21 rosetta2 E. coli strain (Merck Millipore). Cells were lysed by incubation with a 1 mg/ml lysozyme for 30 minutes on ice, followed by sonication. The GST-tagged proteins and His-tagged proteins were purified by affinity chromatography as described (Okiyoneda et al., 2018).

In vitro Ubiquitination assay

RFFL and RNF34 autoubiquitination were measured as done previously (Okiyoneda et al., 2018). On a brief note, either 2 μM GST-RFFL or 2 μM GST-RNF34 were incubated with 0.1 μM His_6-UBE1, 2 μM His_6-sumo-UbcH5c and 20 μM Ub (sigma) in the reaction buffer (20 mM HEPES pH 7.5, 50 mM NaCl, 5 mM $MgCl_2$, 2.5 mM ATP, 2 mM DTT, and 20 mM MG-132) for 2 hours at 30°C and analyzed by a Western blot using either anti-Ub (P4D1, Santa Cruz Biotechnology) or anti-GST (5A7, FUJIFILM) antibodies. NBD1 and Luc ubiquitination were also measured as done previously (Okiyoneda et al., 2018). 250 ng of purified His_6-sumo-NBD1-ΔF1S or 200 ng of Luciferase (Luc, sigma) were mixed with 0.1 μM His_6-UBE1, 2 μM His_6-sumo-UbcH5c, 20 μM Ub, 2 μM GST-RFFL or 2 μM GST-RNF34 in the reaction buffer. The reaction mix was incubated at 30°C for 2 hours after a 5 min incubation at 44°C (NBD1) or 43°C (Luc) to induce the thermal unfolding. The ubiquitination reaction was stopped by adding a SDS sample buffer and analyzed with a Western blot using anti-NBD1 (CFTR 660) and anti-Ub (P4D1) antibodies.

AlphaLISA

112.5 nM His_6-sumo-NBD1-ΔF1S denatured at 44°C for 5 min was incubated with either 75 nM GST-RFFL or 75 nM GST-RNF34 for 2 hours at room temperature in 384 well plates (Perkin Elmer). Then, Ni Donor beads (Perkin Elmer) and Glutathione alphaLISA acceptor beads (Perkin Elmer) were added and incubated for 1

hour in the dark according to the manufacturer's instructions. The direct interaction was detected using the EnSpire Alpha plate reader (Perkin Elmer).

Cell culture and transfection

COS7, GripTite 293MSR (293MSR), HeLa-ΔF508-CFTR-3HA, CFBE-teton-ΔF508-CFTR-3HA, CFBE-teton-ΔF508-CFTR-HRP and CFBE-teton-ΔF508-CFTR-3HA/YFP-H148Q/I152L/F46L cells were cultured as done previously (Okiyoneda et al., 2018). BEAS-2B cells were obtained from The European Collection of Authenticated Cell Cultures (ECACC). BEAS-2B-ΔF508-CFTR-HiBiT, BEAS-2B-teton-WT CFTR-3HA-NLuc (Ct) and BEAS-2B-teton-ΔF508-CFTR-3HA-NLuc (Ct), 293MSR-HBH-ΔF508-CFTR, 293MSR-teton-ΔF508-CFTR-3HA-NLuc (Ct) cells were generated by a lentivirus transduction as done previously (Okiyoneda et al., 2018). ΔF508-CFTR-HiBiT (Ex) was constructed by replacing the 3HA tag in the CFTR (Okiyoneda et al., 2018) to HiBiT. To construct the WT- and ΔF508-CFTR-NLuc, NLuc was fused to the C-terminus of CFTR. Transient expression of plasmids was accomplished using polyethylenimine Max (Polysciences Inc). siRNA transfection (50 nM) in CFBE and BEAS-2B cells was accomplished using the Lipofectamine RNAiMax transfection reagent (Invitrogen). siRNA transfected cells were used for the experiments 5 days post-transfection.

Establishment of RNF34 KO cells by CRISPR/CAS9

The RNF34 KO 293MSR and RNF34/RFFL DKO 293MSR cells were established as done previously (Sakai et al., 2019) using the following gRNA in 293MSR and RFFL KO 293 MSR cells, respectively. RNF34 gRNA #1 (5′-GGCTCCGAACACTTCTTAAT-3′), RNF34 gRNA #2 (5′-CACAATGCTTAGAATGTCGT-3′). The RFFL/RNF34 double KO 293MSR cells were established by a transfection of the two RNF34 gRNAs (#1, #2) to RFFL KO 293MSR cells (Sakai et al., 2019). The RNF34 KO was confirmed by a western blot and genome DNA sequencing. For the sequencing of RNF34, the genomic locus was amplified by PCR using a FW primer 5′-GTCCCCAGTACCTGCATTTGATATG-3′ and a RV primer 5′-GGGAGGGTGCACACCTAGACACCAT-3′. The PCR product was cloned into pMD20-T using a Mighty TA-cloning Kit (Takara Bio) and further determined by DNA sequencing.

Immunocytochemistry

Transfected cells grown on coverslips were fixed in 4% paraformaldehyde (PFA) for 20 min at room temperature (RT) and incubated with DAPI for 5 min at RT, thus mounted in VECTASHIELD mounting medium (VECTOR Laboratories). The fixed cells were incubated with WGA-Alexa Fluor 594 (Thermo) for 10 min for WGA staining. Organelle markers mCherry-Sec61 (Addgene #49155), mRFP-Rab5 (Addgene #14437), mRFP-Rab7 (Addgene #14436), DsRed-Rab9 (Addgene #12677), DsRed-Rab11 (Addgene #12679), and Lamp1-RFP (Addgene #1817) were transiently transfected using PEI Max. Single optical sections were collected on an inverted laser confocal fluorescence microscope (SP8, Leica) equipped with an HC PL APO 63×/NA 1.40 objective. Images were processed with Photoshop CS6 (Adobe). Colocalization of ΔF508-CFTR–GFP with RNF34-mCherry or RFFL-mCherry were analyzed by means of Pearson's correlation coefficient using Volocity 5 (PerkinElmer).

Measurement of PM expression of CFTR

PM density of ΔF508-CFTR-HRP was measured as done previously (Phuan et al., 2014; Veit et al., 2014). For low-temperature rescues, CFBE and BEAS-2B cells were incubated at 26°C and 30°C, respectively, for 2 days followed by a 1 h incubation at 37°C to induce unfolding. PM density of ΔF508-CFTR-HiBiT was measured in 96 well plates using the Nano Glo HiBiT Extracellular system (Promega). For the Trikafta rescue, cells were incubated with 3 μM VX-661, 3 μM VX-445 and 1 μM VX-770 at 37°C for 24 hours (293MSR cells) or 48 hours (CFBE and BEAS-2B cells). BEAS-2B-ΔF508-CFTR-HiBiT cells were treated with 2 mM sodium butyrate (NaB) for 2 days before analysis. The luminescent signal was measured using the Luminoskan and Varioskan Flash microplate reader (ThermoFisher).

Western Blotting

Cells were solubilized in a RIPA buffer supplemented with 1 mM PMSF, 5 μg/ml leupeptin and pepstatin A) where the cell lysates were analyzed by a Western blot as done previously (Okiyoneda et al., 2010).

Ub ELISA

The K48- and K63-linked poly-ubiquitination of mature HBH-ΔF508-CFTR-3HA was measured by the ELISA assay as done previously (Okiyoneda et al., 2018; Kamada et al., 2019). The immature ΔF508-CFTR was minimized by the CHX treatment at 37°C for 3 hours after 26°C rescue (2 days) or Trikafta (3 μM VX-661, 1 μM VX-445, 1 μM VX-771) treatment at 37°C for 2 days. Linkage-specific CFTR ubiquitination levels were normalized to the CFTR levels quantified by an anti-HA antibody (16B12, BioLegend).

Halide-sensitive YFP quenching assay

The ΔF508-CFTR function assay by halide-sensitive YFP fluorescence quenching was performed as described previously (Okiyoneda et al., 2018). PM expression of ΔF508-CFTR in CFBE-teton-ΔF508-CFTR-3HA/YFP-H148Q/I152L/F46L cells were induced by treatment of Trikafta (3 μM VX-661, 0.3 μM VX-445, 1 μM VX-770) for 2 days at 37°C. The YFP fluorescence was measured using a Varioskan Flash (ThermoFisher) with a dual syringe pump (excitation/emission 500/535 nm). The fluorescence was recorded continuously (200 ms per point) for 3 s (baseline) and for 32 s after rapid addition of 100 mL PBS-iodide, in which NaCl was replaced with NaI. Quenching rates were calculated by fitting the YFP fluorescence decay with a one-phase exponential decay function using GraphPad Prism 8 (GraphPad Software).

NanoLuc degradation assay

293MSR teton ΔF508-CFTR-3HA-NLuc cells were seeded onto 96 well white plates and treated with 1 μg/ml of doxycycline (Dox) and Trikafta (3 μM VX-661, 1 μM VX-445, 1 μM VX-770) for 2 days to induce the PM expression of ΔF508-CFTR. 100 μg/ml of Cycloheximide (CHX) was treated at 37°C for 3 hours to minimize the immature ΔF508-CFTR during Nano-Glo Endurazine substrate loading in CO_2 independent medium (ThermoFisher) according to the manufacturer's instructions. After 3 hours of loading, the CFTR-NLuc luminescence was recorded continuously (5 min per point) at 37°C for 10 hours using a Luminoskan microplate reader (ThermoFisher). The half-life of ΔF508-CFTR-NLuc was calculated by fitting with a one-phase exponential decay function using GraphPad Prism 8 (GraphPad Software).

mRNA isolation and q-PCR analysis

The mRNA isolation and q-PCR was performed as described previously (Okiyoneda et al., 2018). The following primers were used; RFFL FW primer 5'-CAAGAGGAACCCGTCTACCTG-3', RFFL RV primer 5'-CACTGTCAGGCCTTCAATGTC-3', RNF34 FW primer 5'-CCCACCAGCAGCTACGGA-3', RNF34 RV primer 5-GGCGCTGAAATGCTGTCTC-3', GAPDH FW primer 5'-CATGAGAAGTATGACAACAGCCT-3', GAPDH RV primer 5'-AGTCCTTCCACGATACCAAAGT-3'. CF Human bronchial epithelial cells (CF-HBE) and WT-HBE were purchased from the Cystic Fibrosis Translational Research center (CFTRc), McGill University. CF-HBE and WT-HBE were expanded using conditional reprogramming (Avramescu et al., 2017) followed by differentiation on filter supports for more than 4 weeks following established protocols (Neuberger et al., 2011).

Statistical analysis

For quantification, data from more than two technical repeats for each independent experiments were used where the data is expressed as means ± SE. Statistical significance was assessed by either a two-tailed paired Student's t-test or a one-way ANOVA using GraphPad Prism 8 (GraphPad Software).

The supplemental Figures

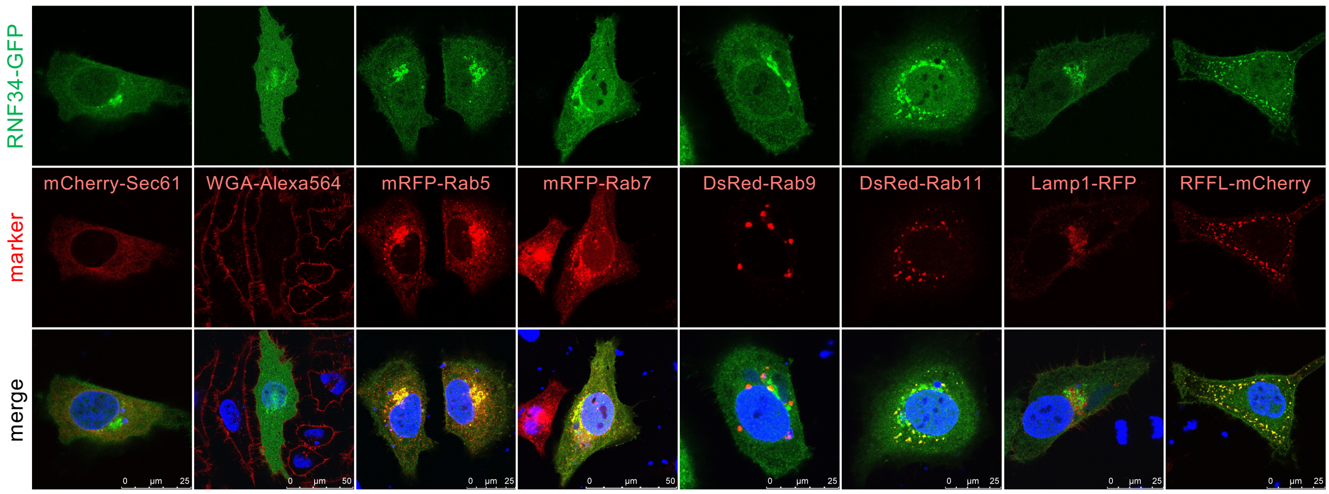

FIGURE S1. Related to FIGURE 2.
Split images of the merged fluorescence micrographs are shown in the FIGURE. 2A.

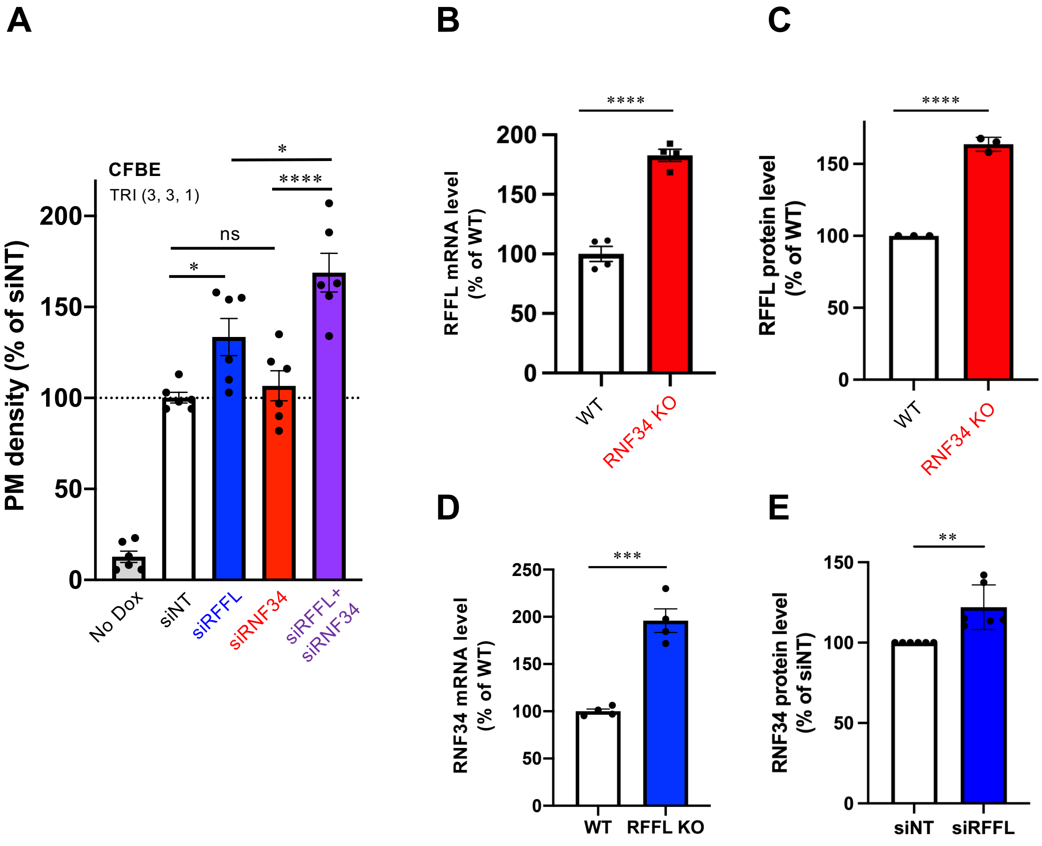

FIGURE S2. Related to FIGURE 3.
(A) PM density of ΔF508-CFTR-HRP in CFBE transfected with siRNA are indicated. Cells were treated with Trikafta (3 μM VX-661, 3 μM VX-445, 1 μM VX-770) for 48 hours. (n=6). **(B, C)** RFFL mRNA (B, n=4) and protein levels (C, n=3) in 293MSR (WT) and RNF34 KO cells were measured by qPCR and a Western blot, respectively. **(D)** RN34 mRNA level in 293MSR (WT) and RFFL KO cells were measured by qPCR (n=4). **(E)** RNF34 protein levels in BEAS-2B-ΔF508-CFTR-HiBiT cells transfected with either siNT or siRFFL were measured by Western blotting (n=6). Statistical significance was assessed by either a one-way ANOVA (A) or a two-tailed paired Student's t-test (B, C, D, E). Data represents mean ± SE. *p<0.05, **p<0.01, ***p<0.005, ****p<0.001, ns, not significant.

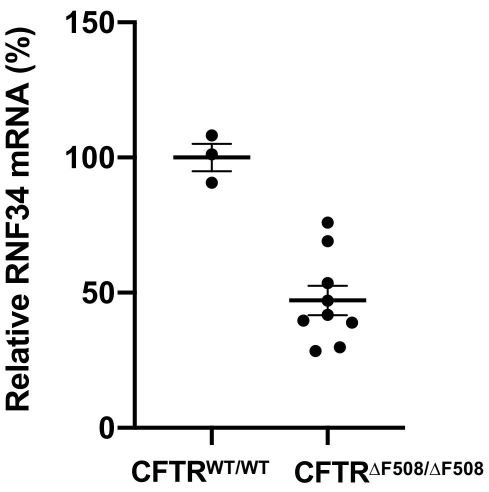

FIGURE S3.
The RNF34 mRNA expression in differentiated human bronchial epithelial cells (HBE) isolated from three donors with $CFTR^{WT/WT}$ and nine patients with $CFTR^{\Delta F508/\Delta F508}$ genotype was measured by q-PCR. The HBE cells were differentiated on filter supports under air-liquid interface culture conditions for ≥ 4 weeks. Error bars and horizontal lines show SE and means of all data, respectively.
